# TNF-*α* and Microalbuminuria in Patients with Type 2 Diabetes Mellitus

**DOI:** 10.1155/2014/394206

**Published:** 2014-12-22

**Authors:** I.-Th. Lampropoulou, M. Stangou, A. Papagianni, T. Didangelos, F. Iliadis, G. Efstratiadis

**Affiliations:** ^1^Department of Nephrology, “Hippokratio” General Hospital, Aristotle University of Thessaloniki, 49 Konstantinoupoleos Street, 546 42 Thessaloniki, Greece; ^2^1st Propedeutic Department of Internal Medicine, Diabetes Center, “AHEPA” General Hospital, Aristotle University of Thessaloniki, 546 36 Thessaloniki, Greece

## Abstract

*Aim.* Recent evidence suggests that chronic subclinical inflammation plays a key role in the pathogenesis and progression of diabetic nephropathy. Aim of the present study was to investigate possible correlation between the presence and degree of microalbuminuria and markers of inflammation in patients with type 2 diabetes mellitus (DM).* Patients-Methods.* Eighty patients were enrolled and clinical and laboratory data were recorded. Albumin-creatinine ratio (ACR) was calculated in first-morning urine samples. Serum and urinary tumor necrosis factor-*α* (TNF-*α*) levels were determined by ELISA.* Results.* Forty-five patients had normoalbuminuria, 33 microalbuminuria, and 2 macroalbuminuria. Patients with microalbuminuria were older, with higher glycosylated hemoglobin levels (HbA1c) and they more frequently had diabetic retinopathy, neuropathy, and cardiovascular disease and were on treatment with angiotensin converting enzyme inhibitors (ACEi) and/or angiotensin receptor blockers (ARBs). ACR was significantly correlated with the presence of cardiovascular disease, hypertension, and HbA1c levels and the administration of clopidogrel and ACEi or ARBs. ACR was not correlated with C-reactive protein, fibrinogen, or serum TNF-*α* levels but had a strong correlation with urinary TNF-*α* levels.* Conclusions. *In patients with type 2 DM, urinary, but not serum, TNF-*α* levels are associated with the presence and severity of microalbuminuria.

## 1. Introduction

Diabetic nephropathy, the major microvascular complication of diabetes mellitus (DM), has been widely considered as the central cause of morbidity and mortality in diabetic patients [1, 2]. Until recently, diabetic nephropathy has been exclusively attributed to the interaction between metabolic parameters (activation of polyols [3, 4], exozamines [5], diacylglycerols [4, 6], and advanced glycosylated end products-AGEs [7–11]), hemodynamic factors (systemic hypertension, intraglomerular hemodynamic changes, angiotensin II [12], endothelin I [4]), and oxidative stress [13], governed by genetic and environmental factors. However, recent evidence suggest that chronic subclinical inflammation may play a key role in the initiation and progression of diabetic nephropathy [14]. Tubulointerstitial infiltration by inflammatory cells is evident in renal biopsies from patients with diabetic nephropathy. Furthermore, several metabolic and hemodynamic factors and oxidative stress can regulate cell signalling pathways on glomerular and tubulointerstitial cells, by activating protein kinase cascade and transcription factors, such as nuclear factor-*κ*B (NF-*κ*B) [14, 15]. This activation results in the increased production of cytokines, chemokines, and growth factors and the upregulation of adhesion molecules and leads to a vicious cycle between activated leucocytes that migrate to the interstitial space and produce preinflammatory molecules which activate further native and inflammatory cells. All the above finally leads to the development of glomerulosclerosis and tubulointerstitial fibrosis between infiltrating cells and inflammatory molecules [14–16].

Tumor necrosis factor-*α* (TNF-*α*) is produced by activated native kidney cells (glomerular mesangial, epithelial and endothelial cells, and tubular epithelial cells) and also by activated monocytes-macrophages [14, 16] and increases the release of other cytokines, chemokines, growth factors, and acute phase proteins [14]. As a pleiotropic cytokine, TNF-*α* exerts multiple effects and it can contribute to the development of diabetic nephropathy through several mechanisms, including reduction of the glomerular blood flow and glomerular filtration rate, vasoconstriction induced by increased endothelin-1 production, and disruption of the glomerular filtration barrier which is mediated by the interaction with the intercellular junctions and leads to proteinuria [14]. Increased production of TNF-*α* can also produce oxidative stress, through the activation of nicotinamide adenine dinucleotide phosphate, (NADPH), in mesangial cells. Finally, TNF-*α* appears to have a direct apoptotic and cytotoxic effect on glomerular cells [14, 17].

Despite extensive research, there are still unexplored areas and unanswered questions regarding the role of inflammation in the pathogenesis of renal damage in diabetes mellitus [17–21] and furthermore the implication of TNF-*α* in the initiation of inflammatory cascade. The aim of the present study was to investigate possible associations between the presence and degree of microalbuminuria, as the first indications of diabetic nephropathy and the markers of inflammation in patients with type 2 DM.

## 2. Patients-Methods

### 2.1. Patients

Eighty patients with type 2 DM, followed up at the Outpatient Clinic of the 1st Department of Internal Medicine of “AHEPA” University Hospital in Thessaloniki Greece, consecutively entered the study. Patients with liver disease, autoimmune disease, or malignancies were excluded in order to avoid the possible effects of these comorbid conditions on cytokine production. None of the patients was receiving antibiotics, nonsteroidal anti-inflammatory drugs, corticosteroids, or cytotoxic drugs at the time of the study.

Patients' demographic, anthropometric, and clinical characteristics and the administered drugs were recorded. The presence of diabetic neuropathy was estimated by the Michigan Neuropathy Screening Instrument (MNSI) protocol. The presence of diabetic retinopathy was tested by fundoscopy, which was performed within six months before study entry. Cardiovascular disease was defined as the presence of coronary artery disease (medical history of myocardial infarction or revascularization), cerebrovascular disease, abdominal aortic, or lower extremity arterial disease.

Body mass index (BMI) was calculated by dividing the body weight in kilograms (Kg) by the square of the height in meters (m). Estimated glomerular filtration rate (eGFR) was calculated using the Cockroft-Gault formula, eGFR = [(140 − age)∗body  weight]/72∗serum  creatinine, and ∗0.85 if women. eGFR was expressed in mL/min/1.73 m^2^. Systolic (SBP) and diastolic (DBP) blood pressure were measured with a mercurial manometer, for three sequential times, after a five-minute rest period, with the patients on backstroke position and the left arm on the same position. Mean blood pressure (MBP) was calculated by the equation MBP = DBP + (SBP − DBP)/3. Hypertension was defined as SBP ≥ 140 mmHg or/and DBP ≥ 90 mmHg or the regular use of antihypertensive drugs. Smoking was defined as current use of tobacco products or use up to six months prior to study entry.

The study was approved by the Hospital's Institutional Review Board and informed consent was obtained by all patients prior to study entry.

### 2.2. Methods

Blood samples were taken from a peripheral vein under fasting conditions. Hematology and biochemistry were determined by routine techniques using an automated analyser. LDL cholesterol was calculated using the Friedewald formula. Serum C-reactive protein (CRP) levels were evaluated by high sensitivity nephelometry, and exact values were obtained in all patients (normal values < 0.8 mg/dL).

First-morning urine samples were collected under sterile conditions. 10 ml of urine were centrifuged at 1500 rpm for 10 min and the supernatant was stored at −80°C until TNF-*α* assay. The same specimen was used for urinary cultures and for the measurement of albumin-creatinine ratio, ACR (*μ*g/mg). ACR < 30 *μ*g/mg was defined as normoalbuminuria, 30–300 *μ*g/mg as microalbuminuria, and ACR > 300 *μ*g/mg as macroalbuminuria.

Serum samples were separated from clotted blood by immediate centrifugation (1500 rpm for 10 min), aliquoted, and stored at −80°C until TNF-*α* assay.

Serum and urinary TNF-*α* levels were measured by an enzyme-linked immunoabsorbent assay (ELISA) using commercially available standard kits (Quantikine high-sensitivity human TNF-*α* Research & Diagnostic Systems, Europe Ltd, Abington, UK) in the Laboratory of Kidney Disease Research of the Department of Nephrology at “Hippokratio” General Hospital. The lower detection limit was 0.5 pg/mL. Urinary TNF-*α* levels were standardized by the amount of creatinine in the urine and expressed as pg/mg urinary creatinine.

## 3. Statistical Analysis

Data are expressed as mean ± SD, median with range or number of patients (%) as appropriate. The significance of differences in means between groups was assessed by Student's* t*-test or the Mann-Whitney* U* test as appropriate. Differences in proportions were tested with the use of the chi-square statistic. Correlations were tested by regression analysis. Not-normally distributed variables were log-transformed before entering regression analysis. Multiple regression analyses with a forward elimination procedure were used to assess the combined influence of variables on ACR values. The calculations were performed using SPSS 17.0, statistical software. A two-tailed *P* value of 0.05 was considered to be statistically significant.

## 4. Results

The demographic, anthropometric, and clinical characteristics of the patients as well as the prescribed drugs are shown in Table 1.

Median age was 68 years and 60% of the patients were female. Median duration of diabetes mellitus was 14 years and mean eGFR 76.7 mL/min/1.73 m^2^. Sixty-one patients (76.3%) were receiving statins, 46 (57.5%) angiotensin converting enzyme inhibitors (ACEi) and/or angiotensin receptor blockers (ARBs), 21 (26.3%) acetylsalicylic acid, and 25 (31.3%) clopidogrel. Diabetes was controlled with oral hypoglycaemic agents alone in 55 (68.8%) patients, insulin in 4 (5%) patients, and a combination of oral hypoglycaemic agents and insulin in 21 (26.3%) patients.

Laboratory results are shown in Table 2. ACR was 55.89 ± 70.96 *μ*g/mg, CRP was 0.52 (0.13–1.9) mg/dL, fibrinogen was 3.28 ± 0.5 g/L, serum TNF-*α* was 4.28 ± 5.01 pg/mL, and urinary TNF-*α* was 7.12 ± 8.09 pg/mg creatinine.

### 4.1. Correlations of ACR with Clinical Characteristics, Prescribed Drugs, and Laboratory Findings

ACR was significantly correlated with a history of cardiovascular disease (*P* = 0.026), administration of clopidogrel (*P* = 0.012) and ACEi/ARBs (*P* = 0.003), and levels SBP (*r* = 0.24, *P* = 0.05). In addition, ACR had a weak, not significant correlation with duration of diabetes (*r* = 0.18, *P* = 0.09), BMI (*r* = 0.2, *P* = 0.07), and the presence of hypertension (*P* = 0.06) and a borderline correlation with age (*r* = 0.25, *P* = 0.05). No significant association was observed between ACR and the presence of diabetic retinopathy, neuropathy, MBP, and DBP and the administration of statins or acetylsalicylic acid.

When the presence of microalbuminuria was considered as a categorical parameter, we found that patients with micro- compared to those with normo-albuminuria were older (*P* = 0.01) and had longer duration of diabetes (*P* = 0.02) and higher glycosylated hemoglobin levels (*P* = 0.001). In addition, a greater percentage of patients with microalbuminuria had retinopathy (*P* = 0.02), neuropathy (*P* = 0.04), and a history of cardiovascular disease (*P* = 0.02) and were receiving ACEi/ARBs (*P* = 0.001). ACR showed a positive and significant correlation with glycosylated hemoglobin levels (*r* = 0.39, *P* = 0.001) and a weak negative correlation with eGFR (*r* = −0.25, *P* = 0.05). No association was observed between ACR and LDL and HDL-cholesterol and triglyceride levels.

### 4.2. Correlations of ACR with Markers of Inflammation

**Figure 1:**
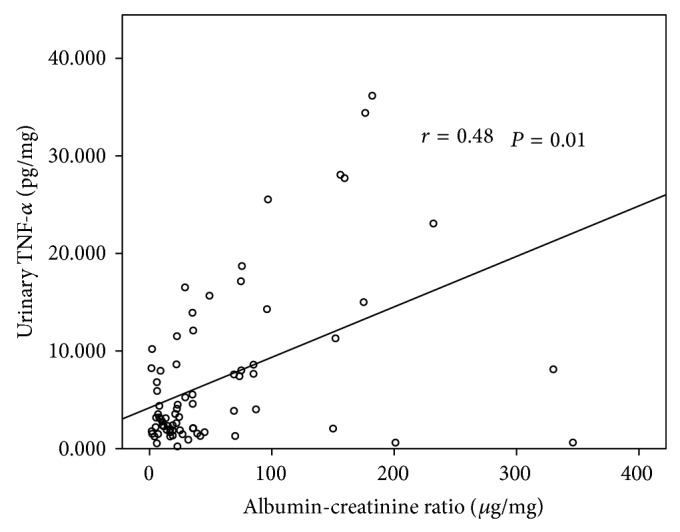
Correlation between ACR and urinary TNF-*α* in patients with diabetes mellitus type 2.

ACR had no significant correlation with CRP, fibrinogen, or serum TNF-*α* levels. In contrast, urinary TNF-*α* levels had a significant positive correlation with ACR (*r* = 0.48, *P* = 0.01) (Figure 1). Multivariate analysis showed that urinary TNF-*α* levels were the only independent contributor to ACR values (*r* = 0.39, *P* < 0.05). Factors included in the multivariate analysis were age, duration of diabetes, BMI, SBP, history of cardiovascular disease, presence of retinopathy, hypertension, glycosylated haemoglobin levels, treatment with clopidogrel, ACEi/ARBs, and eGFR and urinary TNF-*α* excretion. The variables that were entered into the model had a* P* value < 0.1 in univariate analysis. Of note, no association was observed between serum and urinary TNF-*α* levels.

### 4.3. Urinary and Serum TNF-*α* Levels in Patients with Normo- and Microalbuminuria

Forty-five patients had normoalbuminuria (56%), 33 had microalbuminuria (41%), and only 2 patients had macroalbuminuria. The demographic, anthropometric, and clinical characteristics as well as the administered drugs and the laboratory findings of the patients with normoalbuminuria and microalbuminuria are shown in Table 3.

**Figure 2:**
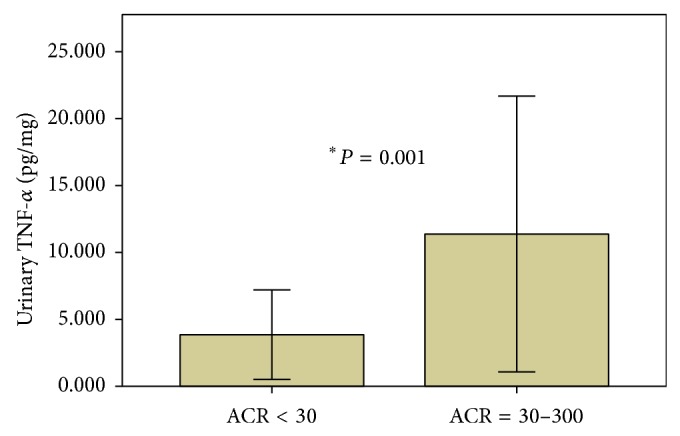
TNF-*α* urinary levels in diabetic patients with normoalbuminuria and microalbuminuria.

Interestingly, compared to patients with normoalbuminuria, patients with microalbuminuria had significantly higher urinary TNF-*α* (*P* = 0.001), (Figure 2), but similar serum TNF-*α* levels. However, there was no correlation between eGFR and serum or urinary TNF- *α*.

## 5. Discussion

The aim of the present study was to investigate the impact of inflammation during the early phase of diabetic nephropathy. Recent evidence suggests an important role of inflammation in the pathogenesis and progression of diabetic nephropathy [14]. The present study investigated the correlation between early markers of diabetic nephropathy, such as microalbuminuria and markers of inflammation in patients with type 2 DM. For this reason, the study population mainly consisted of diabetic patients whose main indication of renal impairment was the presence of microalbuminuria.

Age and duration of diabetes showed a weak positive correlation with the degree of microalbuminuria. Moreover, both parameters were significantly higher in patients with microalbuminuria compared to those with normoalbuminuria. The above results are rather predictable since age and diabetes duration for more than 10 years are well known risk factors for the development of diabetic nephropathy [1, 2]. Furthermore, in our study, the weak negative correlation between degree of microalbuminuria and eGFR may reflect the development of glomerulosclerosis, leading to urine albumin excretion.

The frequency of diabetic retinopathy was significantly increased in the microalbuminuric compared to the normoalbuminuric patients. This finding is consistent with the definition and the natural history of diabetic nephropathy, in which retinopathy usually precedes or appears simultaneously with the development of albuminuria [1, 2].

In the present study, the degree of microalbuminuria was significantly correlated with systolic blood pressure. It is well known that hypertension and diabetes coexist in the majority of patients, and that systolic blood pressure is a major hemodynamic mechanism of proteinuria in diabetic nephropathy [4]. Albuminuria was also significantly associated with the presence of cardiovascular disease and clopidogrel administration. The above reflects the well-known association of albuminuria with generalized endothelial dysfunction and consequently with several vascular complications including cardiovascular.

A statistically significant positive correlation was observed between microalbuminuria and glycosylated hemoglobin levels. Moreover, glycosylated hemoglobin levels were higher in microalbuminuric compared to normoalbuminuric patients. The above findings reflect the association between poor glycemic control and the development of diabetic nephropathy [1, 2]. Our findings are consistent with the results of the UKPDS study (United Kingdom Prospective Study), in which a reduction of glycosylated hemoglobin levels by 0.9% was correlated with a reduction in the relative risk of albuminuria by 30% [22].

Microalbuminuria was associated with the administration of ACEi/ARBs, and, compared to normoalbuminuric, a greater percentage of microalbuminuric patients were on the above drugs. These findings probably reflect the routine prescription of ACEi/ARBs in all diabetic patients with albuminuria due to their well-documented antiproteinuric effect [12].

As mentioned above, recent studies suggest a significant role of inflammation in the pathogenesis and progression of diabetic nephropathy. However, inflammation in diabetic nephropathy is activated by metabolic, biochemical, and haemodynamic disorders which progressively and persistently lead to kidney injury [23].

Inflammation may promote kidney damage through a variety of mechanisms including monocyte migration, complement activation, platelet function regulation, and clearance of cellular debris from inflamed areas [24]. In our study, no significant correlation was observed between microalbuminuria and classic inflammatory markers, such as CRP and fibrinogen. To the best of our knowledge, only a few studies have investigated the association between albuminuria and markers of inflammation in diabetic patients, with conflicting results. In agreement with our results, Choudhary and Ahlawat found that CRP was higher in patients with macroalbuminuria and was positively correlated with the degree of albuminuria [25], but no significant difference was observed between normoalbuminuric and microalbuminuric patients. In contrast with these results, in the study of Niewczas et al., CRP was increased in microalbuminuric compared to normoalbuminuric patients [26].

Similarly, recent studies have reported higher fibrinogen levels in macro- compared to microalbuminuric patients and controls [27], while Corti et al. [28] found increased CRP and fibrinogen levels in diabetic patients with microangiopathic complications (retinopathy, nephropathy) compared to normal subjects but no difference in inflammatory marker levels between patients with and without microangiopathy. Overall, the above findings are in accordance with the present study and suggest that classic inflammatory markers are mainly increased in patients with overt diabetic nephropathy and macroalbuminuria which is probably associated with systemic activation of the inflammatory response.

Human TNF-*α* is a polypeptide of 157 amino acid residues and a molecular weight of 17 kDa [28]. Human TNF-*α* is a nonglycosylated polypeptide and this may be the main difference with mouse TNF-*α* and with human TNF-*β*. Both TNF-*α* and TNF-*β* act through their receptors, TNF-R1 and TNF-R2, and glycosylated forms of both receptors have been found in the urine [28, 29]. TNF-*α* is produced by monocytes-macrophages and by native renal cells (glomerular endothelial, mesangial and epithelial cells, and tubular epithelial cells) and stimulates them to produce other cytokines, growth factors, and chemokines, such as interleukin-8 (IL-8), monocyte chemoattractant protein (MCP)-1, and macrophage-colony stimulating factor (M-CSF) [30]. TNF-*α* upregulates the expression of endothelial and leukocyte adhesion molecules, which mediate the adhesion of monocytes, lymphocytes, and granulocytes to activated endothelium and their subsequent migration [14, 31]. TNF-*α* may promote renal damage in diabetic nephropathy through several mechanisms. It stimulates the production of endothelin-1 and therefore vasoconstriction and reduction of glomerular blood flow and glomerular filtration rate. It has a cytotoxic effect on glomerular cells and stimulates apoptosis. It disrupts the intracellular junctions of the glomerular filtration barrier and increases its permeability resulting in the development of albuminuria [32]. Urinary TNF-*α* serves as a biomarker of renal inflammation, and urinary TNF-*α* and neutrophil gelatinase-associated lipocalin (NGAL) were predictors of renal impairment in diabetic patients [33].

In our study, microalbuminuria was not significantly associated with serum TNF-*α* levels, while other investigators described higher serum TNF-*α* levels both in macroalbuminuric and in microalbuminuric compared with normoalbuminuric patients [34]. A significant correlation was observed between microalbuminuria and urinary TNF-*α* levels and the latter were higher in patients with microalbuminuria than in those with normoalbuminuria. Interestingly, in the present study, multivariate analysis showed that urinary TNF-*α* was the only independent predictor of the degree of microalbuminuria. Recent studies have shown that TNF-R2 inflammatory pathway is predominantly involved in the progression of albuminuria during the early stages of diabetic nephropathy. Therapeutic strategies aiming for TNF receptor inhibition or TNF production showed improvement of glycemic control, reduction of albuminuria, and improvement of renal function [35–37].

Overall, the above findings suggest an important role of this cytokine in the pathogenesis and progression of diabetic nephropathy. In addition, in the present study, no correlation was observed between serum and urinary TNF-*α* levels suggesting mainly intrarenal production of this cytokine and therefore local and nonsystemic activation of the inflammatory response. The above hypothesis is supported by the lack of correlation between microalbuminuria and CRP, fibrinogen and serum TNF- *α* levels in our patients. However, it should be noted that it remains unclear whether TNF- *α* urinary excretion is a marker of glomerular permeability or reflects tubulointerstitial damage. Although the majority of the patients had normal renal function and accordingly minimal or absent tubulointerstitial changes, further studies are needed with measurements of tubulointerstitial markers such as alpha1-microgloburin and/or NGAL to identify the exact source of renal TNF-*α* production.

Finally, in our patients, no correlation was found between serum or urinary TNF-*α* levels and other microvascular or macrovascular diabetic complications such as diabetic neuropathy and cardiovascular disease indicating an early and important role for this cytokine in the pathogenesis of renal disease. Since the majority of the patients had normo- or microalbuminuria and well-preserved renal function, it appears reasonable to conclude that, during the early stages of diabetic nephropathy, local inflammatory pathways, associated with increased production and excretion of TNF-*α*, are activated and contribute to kidney damage progression. In more advanced stages, a systemic inflammatory response could be activated leading to the development of several other micro- and macrovascular complications.

The present study has some limitations and shortcomings that should be considered. First, patients were classified based on one morning spot urine sample, with no repetitive measurements or timed collection being performed. Moreover, we did not include in our analysis patients with macroalbuminuria as our aim was to investigate initially biomarkers of inflammation during the early stages of diabetic nephropathy. However, it would be very interesting to investigate whether the correlation between TNF-alpha and ACR persists in patients with various stages of diabetic nephropathy. Finally, in the present study, no correlation was observed between eGFR and TNF-alpha levels as the majority of the enrolled patients demonstrated normal eGFR values and it would be also interesting to study the above association across a wide range of eGFR. We are currently recruiting patients with macroalbuminuria and/or renal function impairment in order to investigate the above important issues.

In conclusion, in patients with type 2 diabetes mellitus urinary, but not serum, TNF-*α* levels are associated with the presence and severity of microalbuminuria indicating that its intrarenal production is involved in the pathogenesis and progression of diabetic nephropathy. Further studies are needed to elucidate the exact role of this cytokine in diabetic nephropathy.

## Figures and Tables

**Table 1:** Demographic, anthropometric, and clinical characteristics and prescribed drugs of 80 patients with diabetes mellitus type 2.

Age (years)	68 (38–78)
Sex (male/female)	32/48
Duration of diabetes (years)	14 (1–37)
Body mass index (kg/m^2^)	26.1 (21.4–43.4)
Mean blood pressure (mmHg)	99.4 ± 8.6
Systolic blood pressure (mmHg)	135.5 ± 13.2
Diastolic blood pressure (mmHg)	81.4 ± 9.6
Hypertension	53 (66.3)
Smoking	20 (25)
Diabetic retinopathy	31 (41.9)
Cardiovascular disease	33 (41.3)
Diabetic neuropathy	36 (59)
Statins	61 (76.3)
Acetylosalicylic acid	21 (26.3)
Clopidogrel	25 (31.3)
ACEi/ARBs	46 (57.5)
Oral hypoglecemic agents	55 (68.8)
Insulin	4 (5)
Combination (oral hypoglecemic + insulin)	21 (26.3)

Values are expressed as means ± SD, median with range or number of patients (%) as appropriate.

**Table 2:** Laboratory findings of 80 patients with diabetes mellitus type 2.

Hematocrit (%)	39.19 ± 2.67
Hemoglobin (g/dl)	12.77 ± 1
White blood cells (/*μ*l)	7318 ± 1467
Platelets (/*μ*l)	228338 ± 61585
Fibrinogen (g/L)	3.28 ± 0.5
Glycosylated hemoglobin (%)	6.8 ± 0.6
Glucose (mg/dl)	133 ± 30
Urea (mg/dl)	38 ± 11
Creatinine (mg/dl)	0.99 ± 0.2
Uric acid (mg/dl)	4.7 ± 1
Albumin (g/dl)	4.4 ± 0.3
Total cholesterol (mg/dl)	177 ± 39
LDL cholesterol (mg/dl)	108 ± 28
HDL cholesterol (mg/dl)	46 ± 9.31
Triglycerides (mg/dl)	130 ± 55
eGFR (ml/min/1.73 m^2^)	76.7 ± 26.2
ACR (*μ*g/mg)	55.89 ± 70.96
CRP (mg/dl)	0.52 (0.13–1.9)
Serum TNF-*α* (pg/ml)	4.28 ± 5.01
Urinary TNF-*α* (pg/mg creatinine)	7.12 ± 8.09

Values are expressed as means ± SD or median with range as appropriate.

**Table 3:** Demographic, anthropometric, and clinical characteristics, prescribed drugs, and laboratory findings in diabetic patients with normoalbuminuria and microalbuminuria.

Parameter	Normoalbuminuric	Microalbuminuric
	(*n* = 45)	(*n* = 33)
Age (years)	64 (37–78)	70 (51–78)^*^
Sex (male/female)	17/28 (37.8/62.2)	15/18 (45.5/54.5)
Duration of DM (years)	12 (1–38)	18 (2–35)^**^
Body mass index (kg/m^2^)	27 ± 5.1	28.5 ± 4.5
Mean blood pressure (mmHg)	98.33 ± 8.6	101 ± 8.57
Systolic blood pressure (mmHg)	133.4 ± 13.1	137.9 ± 13.1
Diastolic blood pressure (mmHg)	80.8 ± 9.9	82.6 ± 9.2
Hypertension	23 (51.1)	29 (87.9)
Cardiovascular disease	13 (28.8)	19 (57.6)^**^
Retinopathy	12 (30)	19 (59.3)^**^
Diabetic neuropathy	19 (50)	17 (80.1)^***^
Statins	32 (71.1)	28 (84.8)
Acetylosalicylic acid	12 (26.7)	9 (27.3)
Clopidogrel	9 (20)	14 (42.4)
ACEi/ARBs	18 (40)	27 (81.8)^∧^
Fibrinogen (g/L)	3.3 ± 0.45	3.26 ± 0.67
Glycosylated hemoglobin (%)	6.7 ± 0.7	7.06 ± 0.5^∧^
Total cholesterol (mg/dl)	177.2 ± 38.1	171.7 ± 33.4
LDL cholesterol (mg/dl)	108.5 ± 26.6	104.5 ± 25.9
HDL cholesterol (mg/dl)	46.96 ± 9.1	43.18 ± 9.28
Triglycerides (mg/dl)	125.8 ± 56.9	129.9 ± 46.5
C-reactive protein (mg/dl)	0.53 (0.22–1.9)	0.53 (0.13–0.79)
Serum TNF-*α* (pg/ml)	3.79 ± 5.1	4.99 ± 5.06
Urinary TNF-*α* (pg/mg creatinine)	3.8 ± 3.3	11.4 ± 10.3^∧^
eGFR (ml/min/1.73 m^2^)	81.27 ± 28.4	71 ± 22.69

Results are reported as mean ± SD, median with range or number of patients (%) as appropriate.

^*^ *P* = 0.01, ^**^ *P* = 0.02, ^***^ *P* = 0.04, ^∧^ *P* = 0.001 compared to patients with normoalbuminuria.
